# Changes in inflammatory biomarkers in HCV-infected patients undergoing direct acting antiviral-containing regimens with or without interferon

**DOI:** 10.1371/journal.pone.0179400

**Published:** 2017-06-21

**Authors:** Claudia Mascia, Serena Vita, Paola Zuccalà, Raffaella Marocco, Tiziana Tieghi, Stefano Savinelli, Raffaella Rossi, Marco Iannetta, Irene Pozzetto, Caterina Furlan, Fabio Mengoni, Claudio Maria Mastroianni, Vincenzo Vullo, Miriam Lichtner

**Affiliations:** 1 Department of Public Health and Infectious Diseases, Sapienza University, Rome, Italy; 2 Infectious Diseases Unit, Sapienza University, S. M. Goretti Hospital, Latina, Italy; University of North Carolina at Chapel Hill School of Dentistry, UNITED STATES

## Abstract

**Background and aims:**

Increased levels of chemokine interferon-gamma (IFN-γ)-inducible protein-10 (CXCL10), soluble CD163 (sCD163) and soluble CD14 (sCD14) have been reported in HCV infection. The aim of this study was to compare, sCD163 and sCD14 levels in HCV-infected patients undergoing direct acting antiviral (DAA)-containing regimens with or without interferon (IFN).

**Methods:**

sCD163, sCD14 and CXCL10 were longitudinally measured by ELISA in 159 plasma samples from 25 HCV-infected patients undergoing IFN-based treatment plus telaprevir or boceprevir and 28 HCV infected subjects treated with DAA IFN-free regimens. Twenty-five healthy donors (HD) were included as controls.

**Results:**

At baseline CXCL10, sCD163 and sCD14 levels were higher in HCV-infected patients than in HD. CXCL10 and sCD163 levels were significantly decreased in responder (R) patients who achieved sustained virological response (SVR), with both IFN-based and IFN-free regimens, while they were persistently elevated in non-responders (NR) patients who stopped IFN-based treatments because of failure or adverse events. Conversely, sCD14 levels were apparently unchanged during therapy, but at the end of treatment the levels reached normal ranges. Comparing the two regimens, the extent of CXCL10 reduction was more pronounced in patients undergoing DAA IFN-free therapies, whereas sCD163 and sCD14 reduction was similar in the two groups.

Interestingly, only in IFN-based regimens baseline sCD163 levels were significantly higher in NR than in R patients, while in the IFN-free treatment group also patients with high sCD163 plasma levels obtained SVR. At the end of therapy, even if the biomarkers were largely decreased, their levels remained significantly higher compared to HD. Only in the early fibrosis stages, sCD163 values tended to normalize.

**Conclusions:**

These results indicate that IFN-free regimens including newer DAA induce an early and marked decrease in circulating inflammatory biomarkers. However, the full normalization of biomarkers was not obtained, especially in patients with advanced fibrosis, thus underlying the need for a treatment in the early stages of HCV infection.

## Introduction

Hepatitis C virus (HCV) is a major cause of liver disease worldwide, leading to progressive fibrosis, potential development of cirrhosis and hepatocellular carcinoma (HCC) [1]. For years, the standard of care of HCV chronic infection has been the treatment with interferon alpha (IFNα)-based regimens. The advances in therapy from the use of standard IFNα monotherapy to pegylated IFNα (PEG-IFNα) in combination with ribavirin (RBV) and, then, to first generation protease inhibitors telaprevir (TVR) and boceprevir (BOC) resulted in improvements in the rates of sustained virological response (SVR) [2]. Nevertheless, in the last years, a remarkable success in the management of HCV infection was obtained with the introduction in clinical practice of several all-oral IFN-free direct acting antiviral agents (DAAs). These new drug classes improved SVR rate up to 95–100% of cases, increasing the possibility of HCV clearance [3].

Since IFN-free DAA regimens have specific steps of the virus life cycle as a target, they help clarifying the interaction between HCV and the innate immune response, regardless of the IFNα induced immune modulation [4]. It is well known that sustained inflammation and fibrinogenesis represent the basis of liver damage during chronic HCV infection [5]. This process is associated with increased production of inflammatory chemokines and mediators [6] and a permanent activation of the innate immune system, including natural killer (NK) cells and liver monocytes/macrophages, mostly Kupffer cells [7,8]. Various inflammatory and innate immune activation biomarkers have been studied during chronic HCV infection and are correlated with liver disease progression, as well as with the development of HCV extrahepatic manifestations, such as cardio-cerebrovascular diseases [9–11]. The complex interactions between these inflammatory biomarkers and the outcome of HCV infection have led to studies on the effect of antiviral treatment for HCV. In the pre-DAAs IFN-based treatment era, the analysis of circulating biomarkers improved the understanding of immune responses to HCV infection and showed their usefulness in predicting therapeutic responses [12–14]. Among plasma biomarkers, we studied chemokine interferon-γ-inducible protein-10 (CXCL10), soluble (s) CD163 and sCD14, because they play an important role in the pathogenesis of HCV infection and have been associated with hepatic inflammatory activity and fibrosis stage [15–17]. Nowadays, the evaluation of the effect of newer IFN-free regimens on the temporal dynamics of inflammatory biomarkers represents an area of active investigation.

In the present study, we evaluated the effects of DAA-containing regimens with or without IFN in a cohort of HCV infected patients, on dynamic changes in circulating levels of the following biomarkers of innate inflammation and immune activation: i) CXCL10, an inflammatory chemokine reflecting liver expression of interferon stimulated genes; ii) sCD163, a marker of monocyte/macrophage activation; iii) sCD14, a marker of immune activation and microbial translocation.

## Patients and methods

### Study population

The study was conducted in two out-patient clinics of a single referral center (Sapienza University, Rome). The study population included 53 patients with active HCV infection who were treated according to the current Italian national guidelines for HCV treatment. Patients had no evidence of HIV or HBV infection or decompensated liver disease. Based on treatment response, patients were classified as responders (R) if they reached sustained virological response, defined as undetectable HCV RNA in blood 12 weeks after the end of therapy (SVR12). The subjects who stopped treatment because of virological failure (NRvf) or side effects (NRse) were defined as non responders (NR). Twenty-five healthy donors (48, 32–58 years; 42% male/female) were included as controls.

The study was approved by the Ethics Committee of “Sapienza” University of Rome. All subjects signed a written informed consent before enrolment in the study. Data and plasma samples were collected respecting donor’s confidentiality and privacy.

### HCV-RNA and HCV genotype testing

Plasma HCV-RNA levels were determined by RealTime PCR Roche Cobas TaqMan. HCV genotypes and subtypes 1a and 1b were determined by Abbott RealTi*m*e HCV Genotype II.

### Liver fibrosis assessment

Liver stiffness (measured in kPa) was determined by transient elastography with the use of a Fibroscan machine (Echosens). Advanced fibrosis (F4) was defined as a liver stiffness measure (LSM) greater than or equal to 14.5 kPa, severe fibrosis (F3) was defined as a LSM greater than or equal to 10, mild or no fibrosis (F0-F2) was defined as a LSM less than 10 kPa [18]. The biochemical index FIB-4 was also used to assess liver fibrosis and calculated using Sterling's formula: age [years] × AST [IU/L]/platelet count [expressed as platelets × 10^9^/L] × (ALT^1/2^ [IU/L]).

### Samples handling

Venous blood samples were collected from each patient into EDTA and heparin containing tubes (Becton–Dickinson Systems, San Jose, CA) and the cell-free plasma was stored at -80°C until use. A total of 159 plasma samples were collected from HCV-infected patients and 25 from healthy donors. The patients were scheduled for plasma collection using the following timing: T0 = baseline before therapy; T1 = after 4 weeks of therapy; T2 = 12 weeks after the end of treatment for R patient or the time of failure (stopping rule) for NR subjects.

### Detection of soluble biomarkers

Commercially available ELISA kits were used for the quantitative detection of plasma levels of CXCL10, sCD163 and sCD14 (Quantikine, R&D Systems, Minneapolis, MN), according to the manufacturer’s instructions. To preserve the linearity of the assays, samples containing high concentrations of CXCL10 or sCD163 and sCD14 were diluted with an appropriate amount of calibrator diluent. The reported minimum detectable doses of CXCL10, sCD163 and sCD14 were 1.67 pg/ml, 0.177 ng/ml and 0,125 ng/ml, respectively. All samples were tested in duplicate.

### Statistical analysis

All statistical analyses were performed using GraphPad Prism Software version 5 (Software MacKiev). Values are given as median and ranges. Non-parametric Mann-Whitney test and non-parametric Kruskal-Wallis ANOVA with Dunn's post-test were applied to compare the differences in values. Non-parametric Friedman test with Dunn's multiple comparison test was applied to perform longitudinal analyses. We assessed the correlations between inflammatory and clinical parameters using Spearman’s correlation coefficient (r). All statistical analyses were considered significant with *p*-values less than <0.05.

## Results

### Study population characteristics

The clinical characteristics of the 53 HCV-infected patients receiving anti-HCV therapy are shown in Table 1. Twenty-five subjects were treated with an IFN-based treatment including PEG-IFNα/RBV plus first generation protease inhibitors (TVR or BOC), whereas 28 subjects were treated with IFN-free DAA regimens with or without RBV.

**Table 1:** Study population characteristics.

Characteristics	IFN-based	IFN-free	*P*
	treatment group	treatment group	
	(n = 25)	(n = 28)	
**Age (Years)**	**49 (23–67)**	**61 (43–78)**	**0.0008**
**Male/Female; n (% male)**	**20/5 (80%)**	**25/3 (88%)**	**NS**
**HCV-RNA level, copies x 10**^**6**^**/mL (median,ranges)**	**2.28 (0.07–7.9)**	**0.8 (0.01–12.8)**	**0.0013**
**Genotypes (Treatment)**			
**1a**	**8 (TVR = 3; BOC = 5)**	**7 (SOF+ SIM = 7)**	**NA**
**1b**	**17 (TVR = 10; BOC = 7)**	**14 (SOF+ SIM = 6; SOF+LED = 3; 3D = 4; SOF+DAC = 1)**	**NA**
**2**	**0**	**4 (SOF = 3; SOF+SIM = 1)**	**NA**
**3**	**0**	**1 (SOF = 1)**	**NA**
**4**	**0**	**2 (SOF+SIM = 2)**	**NA**
**ALT level, IU/L (median,ranges)**	**105 (42–288)**	**97.5 (28–407)**	**NS**
**AST level, IU/L (median,ranges)**	**73 (26–280)**	**84 (26–178)**	**NS**
**PLT (109/L) (median,ranges)**	**170 (34–290)**	**138 (67–340)**	**NS**
**Liver Stiffness (kPA)**	**11.6 (1.3–37.4)**	**21 (10.9–45.7)**	**0.0006**
**FIB-4 Index (median,ranges)**	**2.1 (0.5–20.4)**	**3.8 (1.5–11.5)**	**0.002**
**SVR-12; n (%)**	**13 (52%)**	**100%**	**<0.0001**
**Non-responders; n (%)**	**12 (48%)**	**0**	**NA**
**Virologic failure**	**4 (33%)**		
**Side effects**	**8 (67%)**		

Results are expressed in median (range). For continuous variable Mann-Whitney test and for variable dichotomous Chi-Square test were performed. HCV-RNA: hepatitis C virus ribonucleic acid, TVR: Telaprevir, BOC: Boceprevir, SOF: Sofosbuvir, SIM: Simeprevir, LED: Ledipasvir, 3D: Ombitasvir, Paritaprevir, Dasabuvir, DAC: Daclatasvir, ALT: alanine aminotransferase, AST: aspartate aminotransferase, PLT: platelets, SVR: sustained virologic response, NA: not applicable, NS: not significant.

In the IFN-based treatment group, 13 patients (52%) were responders and 12 (48%) non-responders, of which 33% NRvf and 67% NRse. All patients treated with IFN-free DAA regimens were responders, since they reached SVR12.

### Soluble biomarkers plasma levels in treatment groups and healthy controls

**Fig 1:**
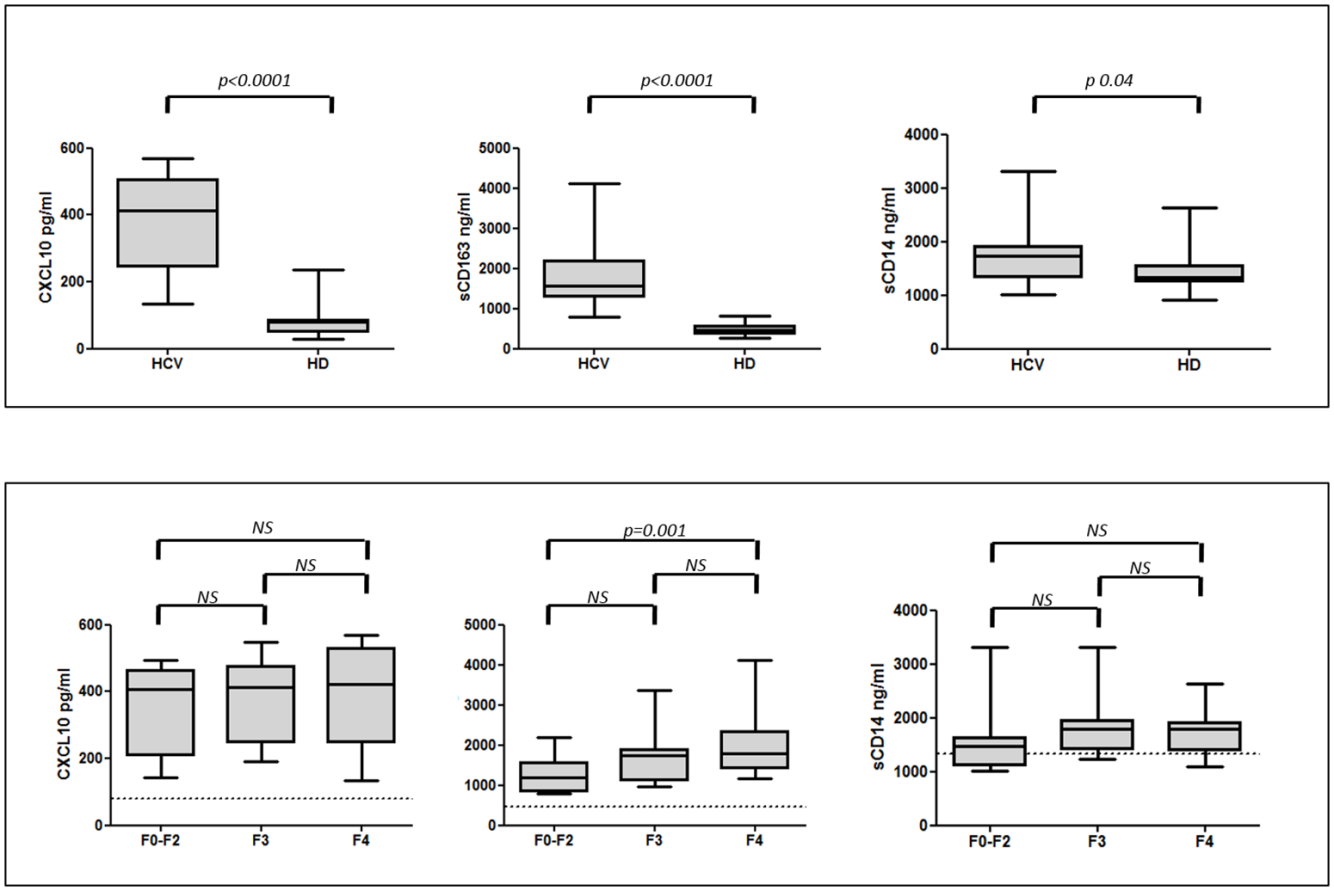
Plasma levels of CXCL10, sCD163 and sCD14 in HCV-infected patients and healthy controls. Panel A: Box plots represent circulating levels of CXCL10 (n = 53), sCD163 (n = 53) and sCD14 (n = 45) in HCV-infected patients at baseline and in 25 HD. Statistical differences were assessed by Mann-Whitney and p are indicated. Panel B: Box plot represents circulating levels of CXCL10, sCD163 and sCD14 in HCV-infected patients according to fibrosis stage at baseline. Horizontal bars represent the median values and dashed lines indicate the median value of HD. Kruskal-Wallis ANOVA with Dunn’s test were performed to assess statistical differences between the different groups. HCV: hepatitis C virus; HD: healthy donors; F2-F0: absent to mild liver fibrosis; F3: severe liver fibrosis; F4: advanced liver fibrosis; NS: not significant.

Plasma levels (median, ranges) of CXCL10 (412.8, 133.8–568.5), sCD163 (1572.6, 790.6–4119.7) and sCD14 (1732.1, 1007.5–3310) in HCV-infected patients at baseline were significantly higher than in HD (81.3, 27.7–236.3; 457.6, 279.2–810.5; 1341.4, 917.4–2627.4, respectively) (Fig 1, Panel A).

When we analyzed the levels of biomarkers according to fibrosis stage at baseline, all groups showed higher levels of CXCL10 and sCD163 in comparison with HD (p<0.001), while sCD14 levels were similar in subjects with F0-F2, F3 liver fibrosis and HD (p>0.05), and were increased only in cirrhotic subjects (p = 0.03) (Fig 1, Panel B).

Patients with severe fibrosis (F4) had significantly higher sCD163 concentrations (1785.7,1169.5–4119.7) compared to those with mild fibrosis (F0-F2) (1194, 790.6–2201.7) (p = 0.001), while no statistically significant differences for CXCL10 and sCD14 levels were found. Moreover, CXCL10 levels positively correlated with FIB-4 (r = 0.4; p = 0.003); sCD163 levels positively correlated with FIB-4 (r = 0.5; p = 0.0004), hepatic stiffness (r = 0.5; p = 0.001), AST (r = 0.4; p = 0.008) and negatively with viremia (r = -0.3; p = 0.03) and platelet absolute counts (r = -0.3; p = 0.02).

### Longitudinal changes in soluble biomarkers during treatment

#### CXCL10 levels

**Fig 2:**
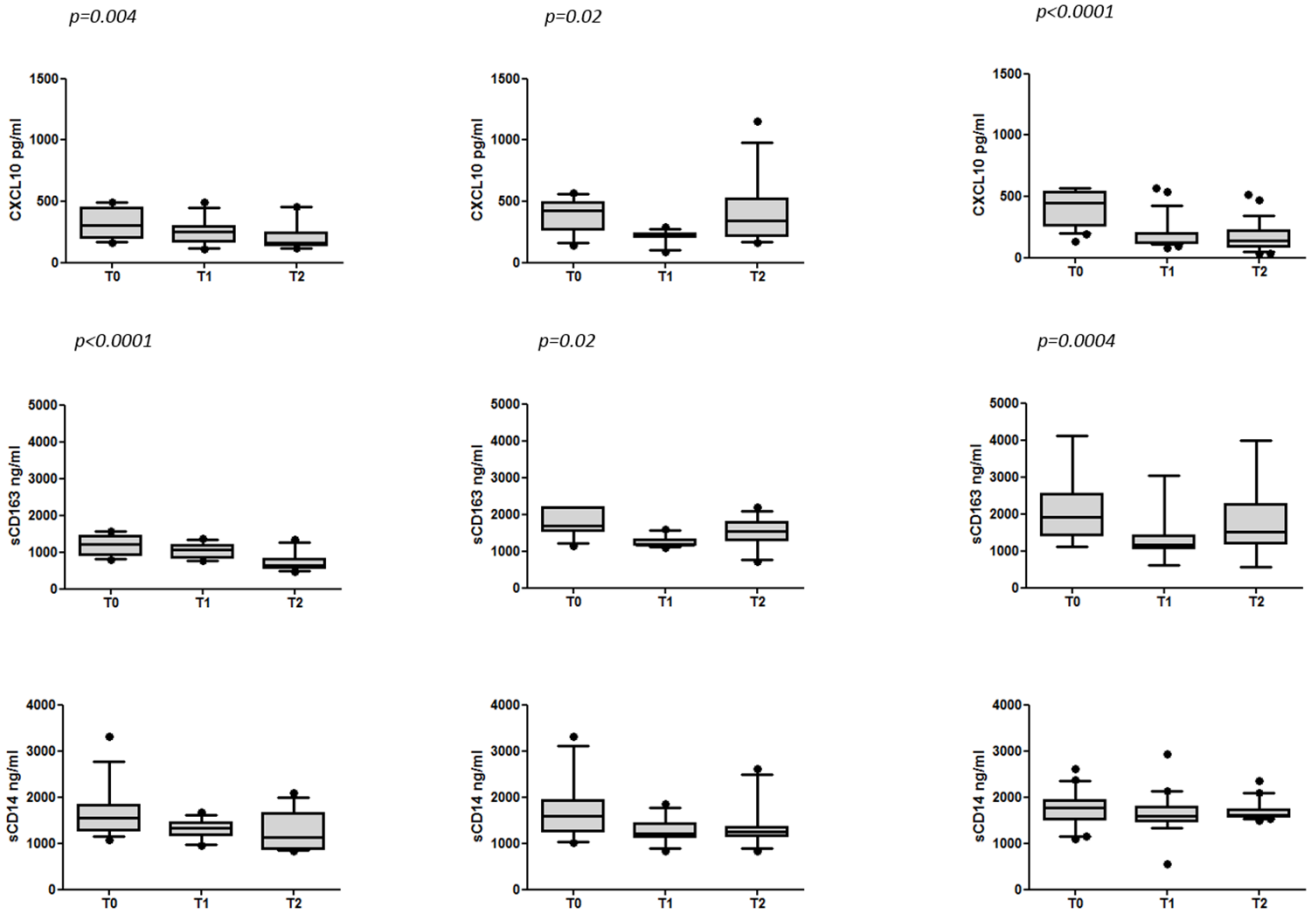
Longitudinal changes in soluble biomarkers in HCV-infected patients undergoing DAA containing regimen with or without IFN. Box plots represent circulating levels of CXCL10 (n = 53), sCD163 (n = 53) and sCD14 (n = 45) in HCV-infected patients at T0, T1 and T2 in R patients during IFN-based treatment, at T0, T1 and T2 in NR patients during IFN-based treatment and at T0, T1 and T2 during IFN-free treatment. Box plots show 10^th^, 50^th^ (median), 90^th^, percentile and whiskers. Horizontal bars represent the median values. Statistical analysis was performed using Friedman test, p values indicate the no post-test. R: responders; NR: non responders; T0: baseline before therapy; T1: after 4 weeks of therapy; T2: 12 weeks after the end of treatment for R patient or the time of failure (stopping rule) for NR subjects.

Changes in the levels of CXCL10 in IFN-based treatment group (R and NR) and IFN-free DAA regimens are shown in Fig 2. At baseline in the IFN-based treatment group, there was not a significant difference in CXCL10 levels between NR (n = 12) and R subjects (n = 13) (p = 0.18). The median (ranges) CXCL10 levels were 426.9 (142.3–568.5) in NR patients and 307.2 (159.9–495.4) in R patients. A significant decrease in CXCL10 concentrations was found one month after initiation of therapy only in R patients and its values remained low up to the SVR12 (T2) (p = 0.004). The median (ranges) value of CXCL10 at baseline was 307.2 (159.9–495.4) and decreased to 249.41 (108.5–495.4) at T1 and 162.4 (115.2–457.8) at T2 (p = 0.004 for T2 versus T0). Conversely, in NR patients, after an initial significant decrease at T1, CXCL10 levels increased at the time of stopping rule. The median (ranges) CXCL10 levels in NR patients were 426.9 (142.3–568.5) at T0, 229.8 (90.1–291.6) at T1 and 345 (164.3–1154.1) at the time of stopping rule (p = 0.02 for T1 versus T0).

In the group of patients treated with IFN-free DAA regimens, there was a marked decrease of CXCL10 plasma levels at T1 and its values remained low up to SVR12 (T2) (p<0.0001). The median (ranges) CXCL10 levels were the following: 451.8 (133.8–568.5) at T0, 123.2 (81.5–568.5) at T1, 137.4 (36–517.6) at T2 (p<0.0001 for both T1 and T2 versus T0).

#### sCD163 levels

The basal sCD163 levels in the IFN-based treatment group were significantly higher in NR patients (median 1702.8, ranges 1139.8–2201.7) than in R patients (1208.1, 790.6–1570.2) (p = 0.0005). During the IFN-based treatment, sCD163 levels significantly decreased in R patients only at T2 (SVR12) (p<0.0001). The median (ranges) sCD163 levels were 1208.1 (790.6–1570.2) at T0, 1062.6 (755.1–1372.1) at T1 and decreased to 635.6 (462.3–1332.1) at T2 (p<0.0001 T2 vs T0). Conversely, in NR patients, after an initial significant decrease at T1, sCD163 levels increased at the time of stopping rule. The median (ranges) sCD163 levels in NR patients were 1702.8 (1139.8–2201.7) at T0, 1178.1 (1098.5–1591.4) at T1, 1546.5 (717.8–2201.7) at the time of stopping rule (p = 0.02 for T1 versus T0) (Fig 2).

In patients treated with IFN-free DAA regimens, sCD163 levels decreased after one month of therapy and its values remained low up to SVR12 (T2) with only a moderate increase (p = 0.0004). The median (ranges) sCD163 levels were the following: 1905 (1124.1–4119.7) at T0, 1161.7 (617.2–3044) at T1, 1524.1 (563.2–4002.3) at T2 (p = 0.0004 for T1 versus T0) (Fig 2).

#### sCD14 levels

Unlike sCD163, there was not a significant difference in sCD14 levels between NR and R subjects at baseline in the IFN-based treatment group (p = 0.76). The median (ranges) sCD14 levels in NR patients were 1602.5 (1007.5–3310) and 1547.5 (1082.5–3305) in R patients.

During the IFN-based treatment, sCD14 levels slightly decreased without statistically significant differences in both R and NR patients. The median (ranges) sCD14 levels were the following in R: 1547.5 (1082.5–3305) at T0, 1335 (956–1677.5) at T1, 1132.5 (842.5–2085) at T2 (p>0.05). The median (ranges) sCD14 levels in NR were the following: 1602.5 (1007.5–3310) at T0, 1214.25 (832.5–1852.5) at T1, 1246 (832.5–2612.5) at T2 (p>0.05). Also in patients receiving IFN-free DAA regimens, there was no significant decrease in sCD14 levels. The median (ranges) sCD14 levels were the following: 1778.9 (1092.5–2612.5) at T0, 1587.8 (552.5–2940) at T1, 1613.3 (1484.3–2347.5) at T2 (p>0.05) (Fig 2).

### Different impact of anti-HCV regimens on soluble biomarkers

**Fig 3:**
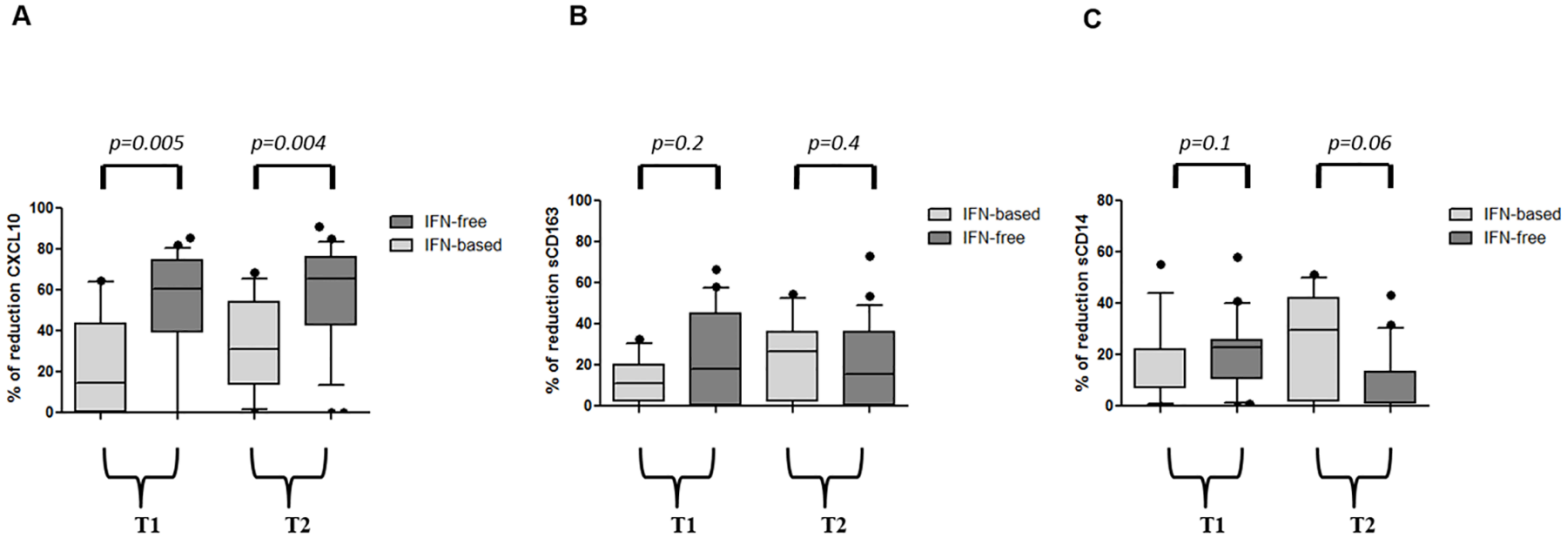
Different impact of anti-HCV regimens on soluble biomarkers. Box plots represent the reduction rate of CXCL10 (panel A), sCD163 (panel B) and sCD14 (panel C) at T1 and T2 versus T0. Box plots show 10^th^, 50^th^ (median), 90^th^, percentile and whiskers. Horizontal bars represent the median values. Statistical differences were assessed between IFN-based versus IFN-free treatment group at T1 and T2, by Mann-Whitney test. T0: baseline before therapy; T1: after 4 weeks of therapy; T2: 12 weeks after the end of treatment for R patient.

In order to assess if IFN-based triple regimen or IFN-free DAA therapy exhibit a different effect on CXCL10, sCD163 and sCD14 levels, we calculated for each biomarker the reduction rate at T1 and T2 versus baseline (Fig 3A–3C). We observed a significant greater reduction rate for CXCL10 during IFN-free therapies, in comparison to IFN-based treatments. CXCL10 reduction rate at T1 and T2 was 60% vs 14% and 66% vs 31%, respectively (Fig 3A); for sCD163 it was 18% vs 11% and 16% vs 27%, respectively (Fig 3B); for sCD14 23% vs 7% and 1% vs 29%, respectively (Fig 3C).

### Biomarkers normalization in HCV-infected patients with sustained virological response

**Fig 4:**
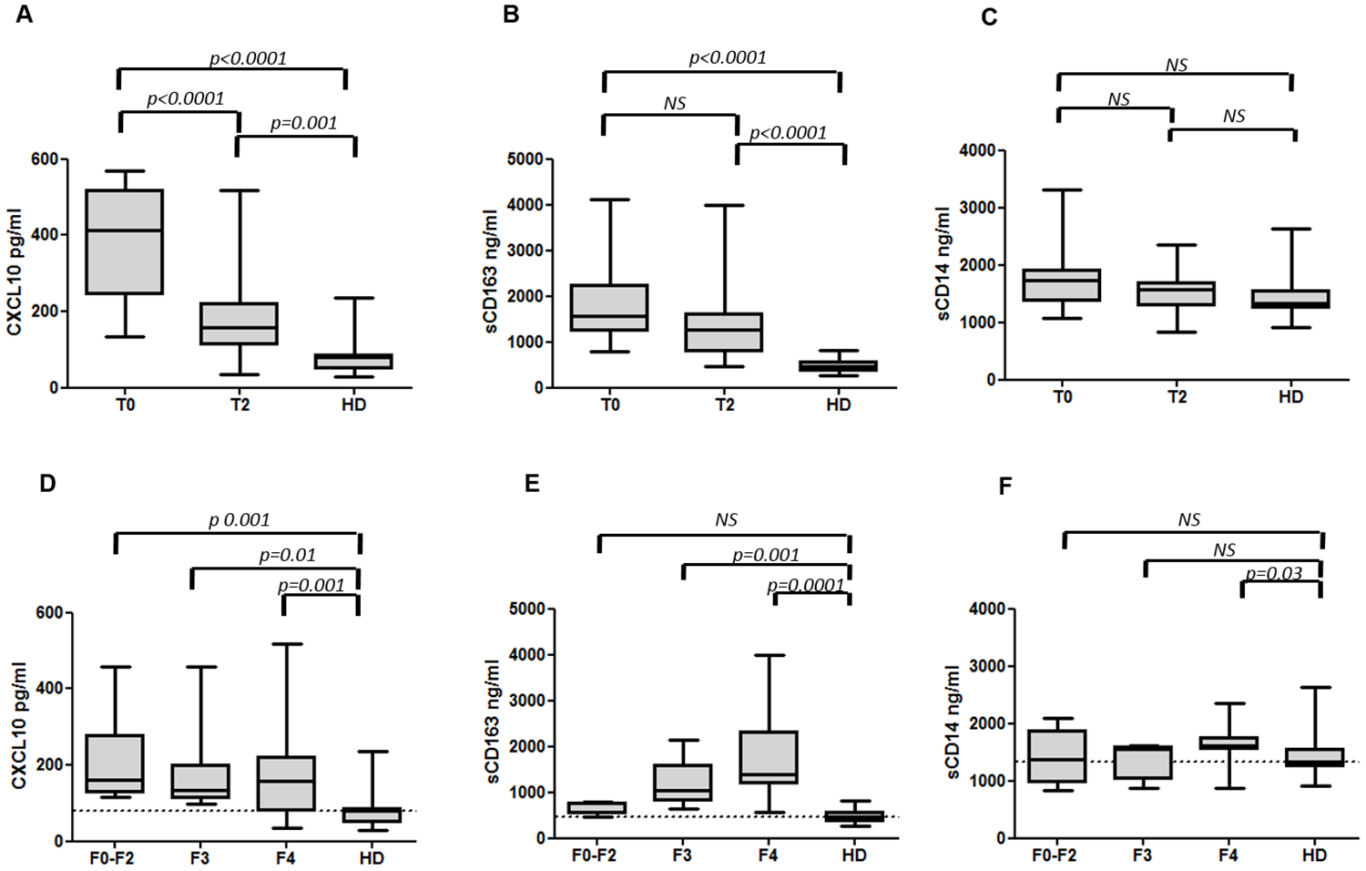
CXCL10, sCD163 and sCD14 normalization in HCV-infected patients with sustained virological response. CXCL10 (panel A), sCD163 (panel B) and sCD14 (panel C) in HCV-infected patients at T0, T2 and in HD. CXCL10 (panel D), sCD163 (panel E) and sCD14 (panel F) in treated HCV-infected patients according to liver fibrosis degree at T2 and in HD. Horizontal bars represent the median values and horizontal dashed line in panel D, E and F indicates the median values of HD. Kruskal-Wallis ANOVA with Dunn’s test were performed to assess statistical differences between the different groups. T0: baseline before therapy; T2: 12 weeks after the end of treatment for R patient; F2-F0: mild to absence of fibrosis; F3: severe fibrosis; F4: advanced fibrosis; HD: healthy donors; NS: not significant.

To assess the degree of normalization of the three biomarkers at SVR12, we compared the levels of CXCL10, sCD163 and sCD14 at T2 (SVR12) with controls HD. Despite a decrease in concentrations, CXCL10 and sCD163 levels did not reach normal values and remained significantly higher than in HD (p = 0.001 for CXCL10 and p<0.0001 for sCD163) (Fig 4A and 4B). On the other hand, sCD14 levels at SVR12 were comparable with those of HD (p>0.05) (Fig 4C).

Interestingly, after stratifying patients according to fibrosis stage, CXCL10 levels remained significantly higher, also in patients with low fibrosis, compared to HD (Fig 4D). Conversely, sCD163 levels decreased in patients with low fibrosis reaching values comparable to those observed in HD (Fig 4E), even if the baseline sCD163 levels were extremely higher than those measured in controls (1194, 790.6–2201.7 and 457.6, 279.2–810.5, p<0.0001). Regarding sCD14, plasma levels remained significantly higher only in cirrhotic patients (Fig 4F), who showed elevated levels also at baseline. Moreover, subjects with F0-F2 and F3 fibrosis, who showed only a moderate increase of sCD14 at baseline, had normal levels in comparison with HD at SVR12.

## Discussion

In this study, we analyzed the temporal modifications of biomarkers of inflammation and immune activation in a cohort of HCV-infected patients undergoing DAA therapies using both IFN-based and IFN-free regimens. The circulating levels of CXCL10, sCD163 and sCD14 were assessed at baseline and during the course of treatment and results were analyzed in terms of virological outcome and response to therapy. As previously reported [19], we found higher plasma concentrations of the three biomarkers at baseline in comparison to controls suggesting systemic immune activation and inflammation, even in the low fibrosis stages of HCV-related disease.

CXCL10 is a member of the CXC subfamily of chemokines induced in monocytes, fibroblasts, and endothelial cells by IFN-γ [4]. After binding to its receptor CXCR3, CXCL10 acts as a chemoattractant for CXCR3+ cells such T lymphocytes, monocytes and NK cells. Intrahepatic and peripheral levels of CXCL10 are elevated in HCV-infected patients with high levels of liver inflammation and fibrosis [20]. The expression of CXCL10 mRNA by hepatocytes correlates with serum CXCL10 levels [21] suggesting that CXCL10 may be a valid surrogate marker of innate immune response activation and of ISG activation in the liver [22]. During HCV infection, T-helper 1 (Th1) lymphocytes secrete cytokines, such as IFN-γ and IL-2, which activate monocytes and macrophages [23]. This immune reaction leads to liver tissue damage and consequent progressive liver disease [24]. In the present study, CXCL10 levels significantly decreased in patients reaching SVR12, suggesting that the decline of this chemokine could be an indicator of disruption of the intrahepatic virus-host interaction. Our study is in accordance with Romero A.I. et al. 2006, who showed a reduction in responders after 6 weeks of IFN/RBV therapy [13]. Indeed, we found a decrease of CXCL10 levels in SVR patients after only one month of therapy, concurrently with HCV suppression. On the other hand, the increased production of CXCL10 in NR patients who stopped treatment for failure or adverse effects may be driven by the rebound of viral replication.

In a recent study, successful treatment with daclatasvir and asunaprevir decreased serum levels of the ISG products CXCL10 and CXCL11, together with a decrease in STAT1 expression and STAT1 phosphorylation in NK cells [25]. In our R patients, we also compared the different impact of the IFN-based and IFN-free regimens on the degree of CXCL10 reduction. Although in R patients the two regimens were both effective in terms of control of HCV replication, a significantly greater reduction of CXCL10 levels was found in the IFN-free than in the IFN-based treatment group. Nevertheless, despite the significant reduction in CXCL10 during treatment, the levels did not reach normal values, suggesting the persistence of residual inflammation. In our study population, we could not observe a significant difference in CXCL10 levels between patients with advanced fibrosis and in those with mild fibrosis, as reported by Diago M. et al. [26], thus underlying the presence of immune activation even in the low fibrosis stages. However, in accordance with Romero A.I. et al [13], we found a positive correlation between CXCL10 levels and fibrosis index (FIB-4), suggesting a possible role of CXCL10 as a non-invasive marker of liver fibrosis.

Furthermore, we studied two markers of monocyte activation, sCD163 and sCD14, which contribute to hepatic inflammation and fibrosis. sCD163 is an important marker of macrophage activation, involved in the activation of hepatic stellate cells during the progression of chronic liver disease [27,28]. In our study, we found higher levels of sCD163 in HCV-infected patients compared to HD, confirming that sCD163 plays an important role in the pathogenesis of HCV infection [29]. In accordance with Kazankow K. et al. [16], we found higher levels of sCD163 in patients with advanced fibrosis (F4) compared to patients with mild fibrosis (F0-F2), showing that sCD163 could play a role in determining the severity of hepatitis. Furthermore, in our study sCD163 levels correlated with fibrosis assessments (FIB-4 and stiffness), suggesting that sCD163 is a promising fibrosis marker during HCV infection [30]. Moreover, the increase in monocyte activation markers has been associated with several cardiovascular and neurodegenerative diseases, such as subclinical carotid artery disease in general population and in HCV or HIV infected subjects [31–34], suggesting a possible extra-hepatic effect of HCV, mediated by monocyte activation.

During anti-HCV therapies, sCD163 levels significantly decreased in R patients undergoing both IFN-based or IFN-free regimens, while they remained high in NR patients who stopped IFN-based treatments for failure or adverse effects. Comparing the two treatment strategies, even if the rate of reduction was lower at T1 in the IFN-based treatment group, no statistically significant differences were observed between the two regimens. Furthermore, in patients who were treated with IFN-based therapy, significantly higher baseline sCD163 levels were observed in NR patients compared to R patients, while in the IFN-free group all patients obtained SVR, including those with higher levels of sCD163 at baseline. Although all patients were treated with DAAs, the response to first generation DAA protease inhibitors was still dependent on the immunomodulating effect of INFα. Comparing inflammation markers reduction rate, we observed a less marked decrease in sCD163 than in CXCL10 plasma levels. Moreover, patients obtaining SVR12 still showed higher levels of sCD163 when compared to healthy subjects. In a recent study sCD163 and Mac2BP levels didn’t normalize within 6 months from the start of interferon-free therapy [35]. Interestingly, in our study, only patients with low fibrosis showed a normalization of sCD163 plasma levels. These findings underline the need to start therapy for HCV infection in an early stage of fibrosis in order to block HCV-induced inflammation. In fact, the persistence of a systemic inflammatory response could be responsible of an increased risk of inflammatory-related organ diseases in HCV infected subjects.

Finally, systemic inflammation associated with gut derived microbial products has been described during HCV infection [17]. sCD14 is a glycosyl phosphatidyl inositol that is expressed on monocytes, macrophages, polymorphonuclear leukocytes and dendritic cells [36]. CD14 is a marker of microbial translocation and is secreted in a soluble form upon monocyte activation. It was demonstrated that sCD14 could be detected at high levels in patients with HCV infections [36] and recent studies suggested a relationship between microbial translocation and progression of liver disease during HCV infection [17]. However, elevated levels of sCD14 have also been observed in patients with non-alcoholic steatohepatitis [37], and common variable immunodeficiency [38], suggesting other possible origins of sCD14 in plasma, one of which may be the liver.

In our study population, we found a moderate increase in sCD14 with a significant increase only in patients with F4 liver fibrosis stage, as described previously [17]. In prior work [39] no significant changes were found in sCD14 levels in patients with HCV infection during PEG-IFNα/RBV therapy, whereas a recent study showed an increase in innate immune activation, measured as sCD14 and IL-18 elevation [40]. In this study the authors showed that a group of patients with low baseline levels of these factors, had a very high SVR rate during the course of dual therapy.

We found that sCD14 levels remained apparently unchanged both in NR and R patients at the end of therapy, despite the control of HCV viremia with no differences between the two therapies. Indeed, when comparing patients who obtained SVR12 with HD, sCD14 levels were similar, but after stratifying patients according to fibrosis stage, cirrhotic subjects showed persistently higher levels of sCD14, indicating a lack of normalization after HCV eradication.

The main limitations of our research are the different clinical characteristics of the two therapeutic groups in terms of liver fibrosis and HCV genotypes due to the distinct indications in the national guidelines at the time of treatment. In fact, current Italian guidelines for HCV treatment allow the use of new DAAs only in patients with advanced fibrosis (F3-F4). Moreover, the number of subjects with low liver fibrosis was limited, together with a short post-treatment follow-up interval.

In conclusion, our results suggest that the systemic levels of inflammatory biomarkers in HCV-infected subjects were globally reduced by anti-HCV therapy, with a more pronounced effect produced by IFN-free regimens on CXCL10 levels. A possible explanation is that CXCL10 declines simoultaneously with the level of viremia and the new and potent therapies with DAAs induce a rapid decrease in HCV replication with a subsequent sudden reduction of CXCL10 plasma levels. This chemokine is induced after RIG-I and TLR-3 activation by HCV RNA, which acts as a PAMP. Furthermore CXCL10 belongs also to the IFN-stimulated gene family [41]. Thus, CXCL10 levels are directly related to viral load, while the other inflammatory markers studied in our work are secondary immune events. In fact, CXCL10 should be considered more linked to viral induced inflammation, while sCD163 and sCD14 more related to secondary immune events that lead to hepatic fibrosis. Moreover IFN-based therapy could induce an immune activation due to the direct effect of IFN that counteracts the anti-inflammatory role linked to HCV suppression.

At the end of therapy, CXCL10 and sCD163 plasma levels remain significantly higher than in HD. Probably the chronic exposure to viral antigens may cause exhaustion of the immune system, resulting in a slow immunological recovery. The low degree of immune recovery could also have important clinical implications that prompt a close monitoring of immune-mediated organ disease associated to with HCV. A recent study suggests that after SVR, T-cell function remains abnormal and hyporesponsive in most cases probably because of cell exhaustion [42]. Only in patients with low fibrosis a normalization of sCD163 was achieved, suggesting the need for an early therapeutic approach during HCV infection. A longer follow-up could clarify if inflammation will resolve over time or persist after HCV eradication.

## Abbreviations

ALT: alanine aminotransferase
AST: aspartate aminotransferase
BOC: boceprevir
CXCL10: chemokine interferon-γ inducible protein 10
HCV: hepatitis C virus
HD: healthy donors
LSM: liver stiffness measure
NK: natural killer
NR: Non Responders
PLT: platelets
R: Responders
RBV: ribavirin
sCD14: soluble CD14
sCD163: soluble CD163
SVR: sustained virological response
TVR: telaprevir
